# MicroRNAs as Biomarkers of Active Pulmonary TB Course

**DOI:** 10.3390/microorganisms11030626

**Published:** 2023-02-28

**Authors:** Galina S. Shepelkova, Vladimir V. Evstifeev, Ruslan V. Tarasov, Anush E. Ergeshova, Mamed A. Bagirov, Vladimir V. Yeremeev

**Affiliations:** Central Tuberculosis Research Institute, Moscow 107564, Russia

**Keywords:** tuberculosis, microRNA, biomarker

## Abstract

The spread of drug-resistant forms of TB dictates the need for surgical treatment in the complex of anti-tuberculosis measures in Russia. Most often, surgical intervention is performed in the case of pulmonary tuberculoma or fibrotic cavitary tuberculosis (FCT). This study is devoted to the search for biomarkers that characterize the course of disease in surgical TB patients. It is assumed that such biomarkers will help the surgeon decide on the timing of the planned operation. A number of serum microRNAs, potential regulators of inflammation and fibrosis in TB, selected on the basis of PCR-Array analysis, were considered as biomarkers. Quantitative real time polymerase chain reaction and receiver operating curves (ROC) were used to verify Array data and to estimate the ability of microRNAs (miRNAs) to discriminate between healthy controls, tuberculoma patients, and FCT patients. The study showed that miR-155, miR-191 and miR-223 were differentially expressed in serum of tuberculoma with “decay” and tuberculoma without “decay” patients. Another combination (miR-26a, miR-191, miR-222 and miR-320) forms a set to differentiate between tuberculoma with “decay” and FCT. Patients with tuberculoma without “decay” diagnosis differ from those with FCT in serum expression of miR-26a, miR-155, miR-191, miR-222 and miR-223. Further investigations are required to evaluate these sets on a larger population so as to set cut-off values that could be applied in laboratory diagnosis.

## 1. Introduction

Granuloma is a special structure in which T-lymphocytes activate infected macrophages, resulting in inhibition of the growth of *Mycobacterium tuberculosis* and a decrease in the rate of infection dissemination. The formation of the central necrotic zone and the outer layers and follicles of lymphocytes complete the maturation of the granuloma. It is important to note that human tuberculous granuloma is characterized by a clear structure with central necrosis surrounded by coaxial layers and follicles of immune cells separating the necrosis from functioning lung tissue. Further increase in inflammation leads to the formation of caseosis and, as a consequence, the formation of caverns. As a result of the collapse of foci of inflammation, the infection begins to spread further through the lymphatic and circulatory systems, an active disease develops and the patient becomes a bacterial excretor and begins to pose a danger to others. Each granuloma behaves as an independent formation. That is, in one patient, both dense granulomas that can control the spread of *M. tuberculosis* and are practically harmless to the host, and caseous granulomas, which are foci of the decay of functional tissues and a place of reproduction of *M. tuberculosis*, can be observed [1].

The basic principle of a unified clinical classification of tuberculosis (TB) is that it is built on the basis of several features: clinical and radiological features of TB forms, the phase of its course and the presence of bacterial excretion. The modern domestic classification of tuberculosis was adopted in 2003 at the 7th Russian Congress of Phthisiologists [2,3].

It consists of four main sections. The first and main part of the clinical classification describes the clinical forms of TB. The second part of the classification depicts the localization of the process in the lungs, the characteristics of the phase of TB process based on clinical and radiological signs and the presence or absence of bacterial excretion, as well as the spectrum of drug resistance of the pathogen. The third part characterizes possible complications. The fourth part characterizes the residual changes after cured TB [2,3].

Fibrotic cavitary pulmonary tuberculosis is a chronic form characterized by the presence of a fibrous cavity, the development of fibrotic changes in the lung tissue and foci of bronchogenic dropout. FCT can result from the progression of any other form of pulmonary tuberculosis. The wall of the chronic cavity consists of three layers: caseous, granulation and fibrous [3,4,5,6].

Tuberculoma of the lung is a clinical form of TB, combining various encapsulated caseous foci of more than 1 cm in diameter, with a long and often low-symptom course. It occurs quite rarely in 3–5% of all TB cases in Russia. Tuberculomas can have various genesis and originate from focal, infiltrative or disseminated TB. A characteristic feature of this form is a prolonged torpid course and the presence of a pronounced connective tissue capsule surrounding large or a group of small foci of caseation and infiltrates. The tuberculoma capsule has two layers: the inner one, adjacent to caseation and consisting of tubercular granulations with epithelioid and giant cells, and the outer one—the fibrous layer. Tuberculoma may stay silent for years, but if progressing can develop severe forms such as pneumonia with caseation, disseminated TB and FCT [3,4,5,6].

These two variants of post primary TB comprise the most common diagnosis at the surgery department of the CTRI. Surgical intervention has been used in TB treatment for a long time. Its importance diminished after the emergence of chemotherapy. However, the spread of rapid multidrug-resistant (MDR) and extensively drug-resistant (XDR) TB has made us return to surgical TB treatment [4,5,6].

For early initiation of adequate chemotherapy, rapid differential diagnosis of the la-tent and active stages of the disease is important. MicroRNAs (miRNAs) might play a major part in the outcome of TB, because early research has implicated miRNAs as regulators of the immune response [7,8,9,10]. MiRNAs are small non-coding RNA molecules, capable of regulating a variety of biological processes, such as cell growth and differentiation, cell metabolism, immune response and inflammation. They affect the expression of genes by interacting with mRNA transcripts, causing their destruction or inhibition of translation [11,12]. The biological significance of the modulation of miRNAs expression in the host’s serum after bacterial infections remains insufficiently clear. Currently, in a number of studies, miRs were shown as useful biomarkers to distinguish between active TB infection and latent tuberculosis infection (LTBI) [13,14,15,16]. However, we were not able to find any publications correlating varying degrees of activity of destructive and “inflammatory” processes in different forms of post-primary pulmonary TB with miRNAs expression levels.

The aim of our study was to assess the miRNAs spectra differences in groups of TB surgery patients and correlate this with the activity and prevalence of TB. In advance of surgery, patients undergo an intensive course of chemotherapy so as to restrict the TB lesion(s) as much as possible. Thus, we attempted to find biomarkers that could help physician to determine the feasibility of the planned surgery.

Here we demonstrated significant changes of some miRNAs serum levels in patients with various forms of post primary pulmonary TB. These changes involved miRNAs from inflammatory and the fibrotic pathways.

## 2. Materials and Methods

### 2.1. Patients and Serum Samples

**Figure 1:**
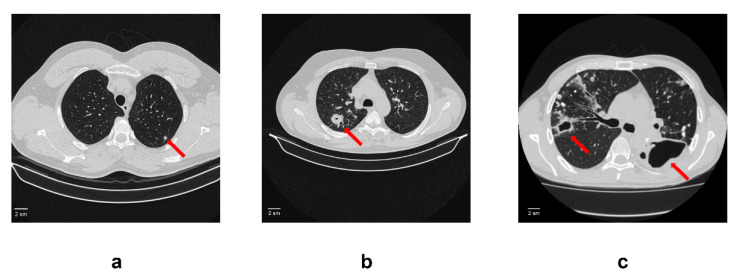
An example of computed tomogram images of active lung TB. (**a**) Lung tuberculoma without “decay”. In S2 of the left lung, there is a rounded formation with clear contours 5 mm in diameter (red arrow); (**b**) Lung tuberculoma with “decay”. In S2 of the right lung, there is a rounded formation 2 cm in diameter with a small decay cavity in the center (red arrow). In the surrounding tissue, single foci and fibrous changes; (**c**) FCT. In the right lung in S2, there is a decay cavity with irregular contours and infiltration around the walls (red arrow). The decay cavity is drained S2. In the rest of the upper lobe, calcifications and foci with infiltration and decay. On the left, there is a large decay cavity in S6 with uneven contours (red arrow). Limited empyema of the pleura on the left in the S6 projection cannot be ruled out.

One hundred and fifty patients with active TB diagnosis aged 18–65 years (69 women and 81 men) were recruited at the surgery department of the Central Tuberculosis Research Institute (Moscow, Russian Federation) between January 2017 and December 2021. The criteria for enrolment were clinical and radiological findings indicating active pulmonary TB including acid-fast bacilli (AFB) growth and/or gene-X pert^®^ positive results. Patients with active TB were divided into 3 groups based on radiological criteria (CT scan data): tuberculoma without “decay” (50 patients), tuberculoma with “decay” (50 patients) and fibrocavernous tuberculosis (50 patients) (Figure 1). Any other infection and complications were set as exclusion criteria for all the study participants. All patients received anti-TB chemotherapy before and after surgery. The duration of preoperative chemotherapy varied from 1 to 3 years.

Fifty healthy age- and gender-matched controls with a negative history of TB disease were also recruited. Individuals without evidence of any clinical TB symptoms or history of TB or any other infections in last three months were included in the control group. All the control subjects were tested for prior exposure to TB using skin immunological tests (Diaskintest^®^, GENERIUM, Moscow, Russia) [17]; the results were negative. Individuals who were on any type of medication were excluded from the study.

All procedures involved and the complete design of the study were in accordance with the Declaration of Helsinki. This study was approved by the Institutional Ethics commit-tee (IEC) of the Central Tuberculosis Research Institute, Moscow, Russia. All participants were informed about the study objectives and procedures, and then consented for collection of specimens (2 mL blood). The patients’ blood was collected not earlier than 1 week prior to surgery.

Blood samples were collected from all subjects and then centrifuged at 1500× *g* for 15 min at 4 °C. Sera were isolated and stored at −80 °C until use.

### 2.2. Total RNA Extraction

miRNeasy Serum/Plasma Spike-in Control (*Caenorhabditis elegans* miR-39 miRNA mimic) (QI-AGEN Gmbh, Hilden, Germany) was added to each serum sample before RNA extraction (3.5 μL miRNeasy Serum/Plasma Spike-in Control per 100 μL serum sample) for monitoring miRNA purification and amplification. An amount of 300 μL of each serum sample was used for RNA extraction. Total RNA extraction from blood serum samples was performed using TRIzol LS (ThermoFisher Scientific (Invitrogen), Waltham, MA, USA), according to the manufacturer’s instructions. The resulting RNA was dissolved into 30 μL of RNase-free water.

Subsequently these RNA samples were used for miScript miRNA PCR Arrays (QI-AGEN Gmbh, Hilden, Germany) and real time PCR (qRT-PCR).

### 2.3. miRNA PCR Array

**Figure 2:**
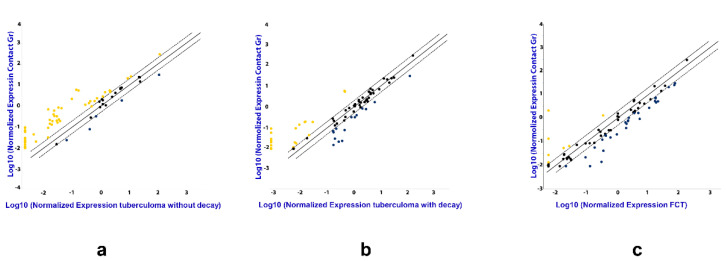
The difference in the expression of genes encoding mature miRNAs in the serum of active lung TB patients. Gene expression values as fold-change for lung tuberculoma without “decay” versus healthy control (**a**), lung tuberculoma with “decay” versus healthy control (**b**) and FCT versus healthy control (**c**). The median value for each gene from three independent replicates per group is presented in log10 scale. Yellow point—upregulated miRNAs as compared with control group; Black point—unchanged miRNAs as compared with control group; Blue point—downregulated miRNAs as compared with control group; Dotted line—the boundaries of the area in which the values are less than 2 times different from the control group; Solid straight line—values are identical to the control group.

The following strategy was used for serum sample screening. Total RNA was purified as described above from TB patients and healthy donors. Out of these, 30 disease samples (for each TB patient group) and 30 healthy donor samples were randomly chosen. The RNA was combined to make 3 pools for each patient group using equal volumes of each RNA sample and 3 donor pools (the same volume as in TB groups), each pool containing RNA from 10 samples. Reverse transcription was performed using miScript II RT Kit (QIAGEN, MA, USA), according to the manufacturer’s instructions, followed by real time PCR quantification, using miScript miRNA PCR Arrays (QIAGEN Gmbh, Hilden, Germany). The data from pooled samples was analyzed using GeneGlobe special software by QIAGEN (Figure 2), and miRNAs assays for further analysis were selected based on our results and literature data.

### 2.4. Quantitative Real Time PCR

Total RNA was reverse transcribed to cDNA with TaqMan^®^ Advanced miRNA cDNA Synthesis Kit (ThermoFisher Scientific (Applied Biosystems), Waltham, MA, USA). The synthesized cDNAs were further used as templates in the qRT-PCR reactions. TaqMan Advanced miRNA Assays (ThermoFisher Scientific (Applied Biosystems), Waltham, MA, USA) were used to analyze miR-191, miR-193a, miR-222, miR-223, miR-320, miR-18, miR-150, miR-155 and miR-26a expression according to the manufacturer’s instructions. miR-103 was chosen as a reference for the data analysis of human samples [18].

### 2.5. Statistical Analysis

GraphPad Prism version 7.0 was used as a statistical tool for miRNA data analysis. Analysis of variance (ANOVA) with multiple comparison was used to compare the fold changes between groups; *p*-values less than or equal to 0.05 were considered significant in all analyses. Data were represented as Mean ± SEM (standard error on mean).

### 2.6. Receiver Operating Characteristics (ROC) Curves

MedCalc version 19.8.0 (MedCalc Software Ltd., Ostend, Belgium) was used to create ROC curves for estimation of the diagnostic value of significantly differentially expressed miRNAs. The areas under the ROC curves (AUC) were used to compare the usefulness of tests. For analyzing the data in our research, we used the following parameters in the ROC curve analysis: AUC values from 90 to 100% were generally considered excellent, 80–90% good, 70–80% fair, 60–70% poor and 50–60% bad (or failed). An AUC below 50% is said to indicate random values not capable of distinguishing between two groups. Thus, the ROC curves were used to evaluate the ability of the fold changes to discriminate between the groups.

## 3. Results

### 3.1. miRNA Expression Analysis

In total, 84 most common (out of about 200 known in human serum) mature miRNAs were examined in miRNA PCR Array.

The analysis by miRNA PCR Array displayed differences in the expression of the miRNAs in TB patients as compared with the control group, and between the three groups of patients with different forms of TB (Figure 2). Patients diagnosed with tuberculoma without “decay” are characterized by increased expression of most of the miRNAs under study compared to the control group (Figure 2a), while patients diagnosed with FCT, on the contrary, have reduced expression compared to the control group (Figure 2c), which may reflect inflammatory reactions accompanying the disease. The weakening of the regulatory influence of miRNAs accompanies the activation of the production of pro-inflammatory factors. At the same time, the tendency towards a decrease in the content of miRNAs in the blood serum is most pronounced in the group of patients with FCT, i.e., where the inflammatory reactions are most pronounced.

Based on the Array data and literature analysis, miR-191, miR-193a, miR-222, miR-223, miR-320, miR-18, miR-150, miR-155 and miR-26a were selected for further investigation with TaqMan RT-PCR assays.

**Figure 3:**
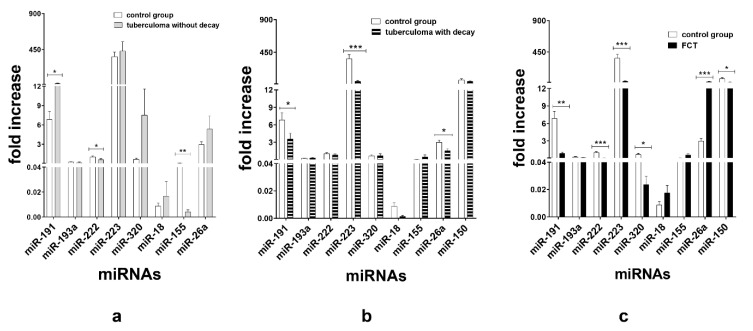
Expression of genes encoding mature miRNAs in the serum of active lung TB patients and control group. Lung tuberculoma without “decay” as compared with healthy control (**a**), lung tuberculoma with “decay” as compared with healthy control (**b**) and FCT as compared with healthy control (**c**). Mean ± SD are shown (n = 50 patients per each group). *—*p* < 0.05; **—*p* < 0.01; ***—*p* < 0.001.

TaqMan assays verified that in the patients with tuberculoma without “decay” group, miR-191 (*p* < 0.05) expression was upregulated and miR-155 (*p* < 0.01) and miR-222 (*p* < 0.05) expression was inhibited as compared with the control group (Figure 3a). In terms of serum miR expression, we observed that the significant difference between the tuberculoma with “decay” patients and the control group was the downregulation of miR-223 (*p* < 0.001), miR-191 (*p* < 0.05) and miR-26a (*p* < 0.05) in tuberculoma with “decay” (Figure 3b). In the FCT, the expression of miR-222 (*p* < 0.001), miR-223 (*p* < 0.001), miR-191 (*p* < 0.01), miR-320 (*p* < 0.05) and miR-150 (*p* < 0.05) was downregulated, and the expression of miR-26a was upregulated, as compared with the control group (Figure 3c), which may reflect the inflammatory reactions accompanying this form of TB.

**Figure 4:**
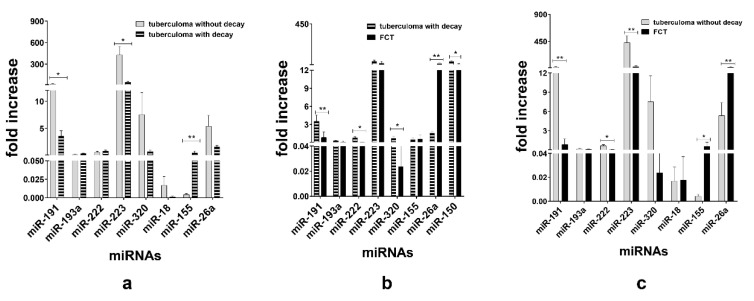
Expression of genes encoding mature miRNAs in the blood serum of active lung TB patients. Lung tuberculoma without “decay” patient’s miRNA expression versus lung tuberculoma with “decay” patient’s miRNA expression (**a**); lung tuberculoma with “decay” patient’s miRNA expression versus FCT patient’s miRNA expression (**b**); lung tuberculoma without “decay” patient’s miRNA expression versus FCT patient’s miRNA expression (**c**). Mean ± SD are shown (n = 50 patients per each group). *—*p* < 0.05; **—*p* < 0.01.

When TB groups were matched, we observed downregulation of miR-191 (*p* < 0.05) and miR-223 (*p* < 0.05) and upregulation of miR-155 (*p* < 0.01) in the sera of the tuberculoma with “decay” patients as compared with those in the sera of the tuberculoma without “decay” patients (Figure 4a). Between the tuberculoma with “decay” and FCT groups, miR-191 (*p* < 0.01), miR-320 (*p* < 0.05) and miR-222 (*p* < 0.05) were significantly upregulated in tuberculoma patients’ sera and miR-26a (*p* < 0.01) in the FCT group (Figure 4b). FCT and tuberculoma without “decay” sera matching revealed downregulation of miR-191, miR-222 and miR-223 in the FCPT group, and miR-26a and miR-155 were more prominent in compared with the tuberculoma without “decay” group (Figure 4c).

### 3.2. ROC Curve Analysis

**Figure 5:**
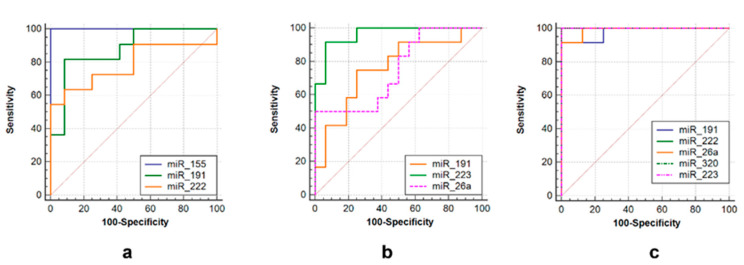
ROC curves showing the diagnostic abilities of (**a**) miR-155, miR-191 and miR-222 to distinguish tuberculoma without “decay” from healthy controls; (**b**) miR-miR-191, miR-223 and miR-26a to distinguish tuberculoma with “decay” from healthy controls; (**c**) miR-191, miR-222, miR-26a, miR-320 and miR-223 to distinguish FCT from healthy controls.

ROC curves were used to assess the diagnostic value of over- or under-expressed miRNAs. Several miRNAs alone could differentiate TB from non-TB controls, such as miR-223 (AUC = 0.958, sensitivity = 92.9%, specificity = 93.7%), miR-191 (AUC = 0.708, sensitivity = 78.6%, specificity = 75%) and miR-26a (AUC = 0.819, sensitivity = 55.3%, specificity = 98.9%) in the tuberculoma with “decay” group (Figure 5b), or miR-191 (AUC = 0.89, sensitivity = 80.0%, specificity = 87.5%), miR-222 (AUC = 0.788, sensitivity = 65.3%, specificity = 87.5%) and miR-155 (AUC = 1.0, sensitivity = 73.0%, specificity = 99.9%) in the tuberculoma without “decay” group (Figure 5a) and miR-223 (AUC = 1.0, sensitivity = 93.7%, specificity = 100.0%), miR-222 (AUC = 1.0, sensitivity = 100.0%, Specificity = 100.0%), miR-26a (AUC = 0.986, Sensitivity = 91.7%, specificity = 100.0%), miR-320 (AUC = 1.0, sensitivity = 100%, specificity = 100%) and miR-191 (AUC = 0.972, sensitivity = 93.3%, specificity = 100%) in FCT patients (Figure 5c).

**Figure 6:**
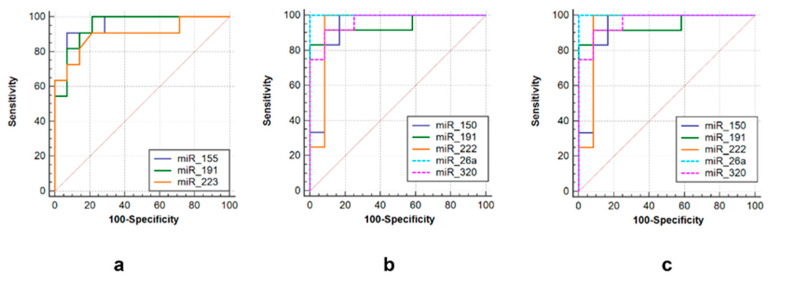
ROC curves showing the diagnostic abilities of (**a**) miR-155, miR-191 and miR-223 to distinguish tuberculoma without “decay” from tuberculoma with “decay”; (**b**) miR-150, miR-miR-191, miR-222 miR-26a and miR-320 to distinguish tuberculoma with “decay” from FCT; (**c**) miR-155, miR-191, miR-222, miR-26a and miR-320 to distinguish tuberculoma without “decay” from FCT.

Three sets of miRNAs were selected to efficiently discriminate between three groups of post primary TB patients. The first one consisted of miR-155 (AUC = 0.948, sensitivity = 90.9%, specificity = 92.9%), miR-223 (AUC = 0.939, sensitivity = 98.2%, specificity = 93.3%) and miR-191 (AUC = 0.948, sensitivity = 86.7%, specificity = 92.9%) to differentiate tuberculoma without “decay” from tuberculoma with “decay” (Figure 6a). The second was comprised of miR-191 (AUC = 0.944, sensitivity = 86.7%, specificity = 85.7%), miR-222 (AUC = 0.938, sensitivity = 93.3%, specificity = 92.3%), miR-320 (AUC = 0.965, sensitivity = 93.3%, specificity = 92.3%), miR-26a (AUC = 1.0, sensitivity = 100%, specificity = 100%) and miR-150 (AUC = 0.931, sensitivity = 93.3%, specificity = 71.4%) to discriminate tuberculoma with “decay” and FCT (Figure 6b). The third one included miR-191 (AUC = 1.0, sensitivity = 100%, specificity = 100%), miR-222 (AUC = 1.0, sensitivity = 93.3%, specificity = 93.3%), miR-223 (AUC = 0.939, sensitivity = 100%, specificity = 93.3%), miR-155 (AUC = 0.977, sensitivity = 100%, specificity = 90.9%) and miR-26a (AUC = 0.932, sensitivity = 100%, specificity = 75%) to discriminate between tuberculoma without “decay” and FCT (Figure 6c).

## 4. Discussion

Here we considered three different forms of active pulmonary TB: tuberculoma without decay, tuberculoma with decay and FCT. Tuberculoma without decay is an inactive form (low inflammation) with a well-formed fibrous capsule and dense caseosis inside. This form of pulmonary TB can be treated with minimally invasive techniques (laparoscopy). Exacerbation of TB leads to the transformation of tuberculoma without decay into tuberculoma with decay (with soft caseosis inside). This is accompanied by perifocal inflammation that spreads to the adjacent lung tissues and leads to an increase in the size of the tuberculoma. Further progress leads to dissemination and dropout foci appearance. The next step is the development of FCT. FCT is characterized by the formation of single or multiple caverns in the lung tissue with widespread fibrosis and foci of dropouts of different origins and prescription as a background. The most common surgical treatment for this form of TB is lobectomy [19].

During the last decade, a number of papers describing serum/plasma miRNAs as TB biomarkers were published. Most studies are devoted to the search for biomarkers of active or latent TB [13,14,15,16]. The standard scheme for such a study includes the identification of the miRNAs expression profile, for example, analysis by the array/microarray method. The use of these methods gives reliable results and makes it possible to identify a large number of candidate genes. However, array screening is generally applied to relatively small groups of subjects (usually no more than 10 subjects). Further, the array results are verified individually in larger groups of patients, using a semiquantitative method—qRT-PCR [13,14,15,16]. In our study we compared the expression of a number of miRNAs in groups of active pulmonary TB patients with varying degrees of active destructive and “inflammatory” processes (tuberculoma without decay, tuberculoma with decay and FCT).

Here we described the changes in expression of certain miRNAs in the serum of TB patients. These changes were dependent on activity TB course. Our results allowed us to define miRNA patterns, capable of differentiating three variants of human active TB from each other and from the control group. The patterns we describe here are based on combinations of seven miRNAs. Destructive variants of TB (FCT and tuberculoma with “decay”) were characterized by downregulation of miR-223 and miR-191.

To date, it is known that miRNAs are involved in the regulation of a number of functions of myeloid cells that determine the outcome of TB infection. The influence of miRNAs on inflammation processes, macrophage polarization, cellular metabolism, autophagy, and apoptosis is known. Along with this, understanding the role of miRNAs in the pathogenesis of human TB is hampered by the specifics of the infection manifestations in different variants of the course of the disease, variability of the pathogen (MDR and XDR-TB) and inability to accurately determine the time elapsed from the moment of infection, etc.

For some of the miRNAs we used in this study, their role in TB infection had been studied to a certain degree. First of all, this may be attributed to miR-155-5p—a key regulator of the innate immunity. Increased expression of miR-155-5p as a result of *M. tuberculosis* infection leads to suppression of the innate immunity at the early stages of infection; however, in general, it plays a protective role against TB, contributing to the survival of infected macrophages and DCs and stimulating their activation and, consequently, recruitment of T lymphocytes, which play a key role in the final control of *M. tuberculosis* through their effector functions [20,21,22].

Infection with *M. tuberculosis* stimulates the influx of neutrophils and macrophages into the lungs, where they contribute to the local inflammation [23,24]. MiR-223-3p plays an important role in the biology of myeloid cells and is widely expressed in the blood and lung parenchyma in human and murine TB [25,26,27]. miR-223-3p controls NF-kB activity in myeloid cells, and negatively regulates cytokines release in TB [24,28]. Ligand 2 of the C-X-C chemokine motif (CXCL2), ligand 3 of the C-C motif (CCL3) and IL-6 are direct targets of miR-223-3p [25]. It should be noted that miR-223-3p knockout mice are unable to control pulmonary TB due to a sharp increase in neutrophil migration and exacerbation of inflammation [25]. Thus, miR-223-3p is able to control excessive inflammation in TB by regulating PMN migration and NF-kB activity.

MiR-26a-5p can attenuate TB-induced host immune response and the IFNγ-dependent activation of macrophages [29]. The target of miR-26a-5p is the p300 transcriptional coactivator, a component of the IFNγ signaling cascade [29]. Contradictory results were obtained in another study [30], which reported a decrease in the expression of miR-26a-5p in macrophages infected with *M. tuberculosis*, as well as in the lungs, spleen and lymph nodes of mice infected with *M. tuberculosis*, while overexpression of miR26a-5p resulted in decreased survival of *M. tuberculosis* in macrophages. Suppression of miR-26a-5p was associated with increased expression of the transcription factor KLF4, which was recognized as a new target for miR-26a-5p [30]. It was demonstrated that KLF4 directs the polarization of macrophages towards the M2 phenotype, characterized by the production of arginase and inhibition of autophagy, and inhibits the transfer of *M. tuberculosis* into lysosomes [30].

Although there are many reports on abnormal miR-191 levels in various forms of cancer, the knowledge of its functional impact is very limited. Studies in breast, colon, gastric and hepatic cancer cell lines suggest that miR-191 is an oncomiRNA [31,32,33,34,35]. miR-191 was demonstrated to be involved in the regulation of cell proliferation, apoptosis and epithelial mesenchymal transition in HCC [33,36,37].

The biological functions and mechanisms of miR-222-3p have been extensively studied recently. Nevertheless, further research on its clinical significance is needed. miR-222-3p is widely involved in the regulation of different cellular, physiological and pathological processes. In addition, expression of miR-222-3p is aberrant during cancer progression [38,39].

miR-320 may be a potential biomarker for metabolic diseases such as obesity, diabetes and its complications, providing a new diagnostic strategy for these diseases. The effects of miR-320 on diseases associated with impaired glucose and lipid metabolism have been described [40].

Zhu et al. discovered that *M. tuberculosis* infection leads to downregulation of miR-18b-5p expression in human macrophage lines [41]. They also demonstrated hypoxia-inducible factor 1α (HIF-1α) to be the target for miR-18b-5p. HIF-1α is known to regulate pro-inflammatory cytokine (IL-6) production through activating MAP-kinases [42]. Because IL-6 and other pro-inflammatory cytokines activate macrophage bactericidal function, miR-18b-5p might regulate the anti-TB effect of human macrophages by promoting an inflammatory response. Nevertheless, we were unable to detect any significant differences in miR-18 expression between both groups of patients with active TB and between TB patients and healthy controls.

## 5. Conclusions

We demonstrated significant changes of certain miRNA serum levels in patients with various forms of active TB. Serums miR-155, miR-191 and miR-223 might be useful for the discrimination of tuberculoma with “decay” from tuberculoma without “decay”. Another combination (serum miR-26a, miR-191, miR-222 and miR-320) forms a set to differentiate between tuberculoma with “decay” and FCT. Finally, tuberculoma without “decay” patients differ from those with FCT in the serum expression of miR-26a, miR-155, miR-191, miR-222 and miR-223. The sensitivity and specificity of the method based on the aforementioned miRNAs as biomarkers of TB activity were 71–100%. Thus, various variants of TB course with different degrees of destruction and intensity of inflammatory processes could be characterized according to the level and direction of expression of the set of miRNAs described here. This set is comprised of seven miRNAs (miR-155, miR-191, miR-223, miR-26a, miR-222, miR-150, miR-320) and might be used during the course of pre-surgery chemotherapy to control TB-induced lung destruction.

## Data Availability

Not applicable.
